# Beyond performance metrics: evaluating the unique value of generative AI in hybrid cybersecurity threat detection

**DOI:** 10.3389/fdata.2026.1768366

**Published:** 2026-04-24

**Authors:** Juan Antonio González-Ramos, Pablo Chamoso

**Affiliations:** 1 Computing and Automation Department, University of Salamanca, Salamanca, Spain; 2 Computing and Automation Department, BISITE Research Group, University of Salamanca, Salamanca, Spain

**Keywords:** cybersecurity, explainable AI, generative artificial intelligence, hybrid systems, large language models, machine learning, threat detection, zero-shot learning

## Abstract

**Introduction:**

This study examines the role of generative artificial intelligence (GenAI) in cybersecurity threat detection, focusing on its usefulness in workflows that support human decision-making.

**Methods:**

Experiments were performed on the BODMAS dataset (134,435 samples) and a smaller exploratory subset of UNSW-NB15. State-of-the-art machine learning (ML) classifiers were compared with a zero-shot large language model (LLM) using standard classification metrics, while also considering latency, cost, and hallucination risk.

**Results:**

ML classifiers consistently outperformed the LLM-based system on standard detection metrics. However, the LLM showed value in cases of ambiguity, where it could provide short plain-language explanations, organize alert-related context, and generate initial interpretations for instances that did not match learned classes.

**Discussion:**

GenAI is unlikely to replace ML-based detection methods, but it can provide useful interpretive support for ambiguous or unfamiliar alerts. A hybrid pipeline is therefore proposed, in which ML handles high-confidence and time-sensitive decisions, while the LLM is used selectively for low-confidence cases or when explanatory support is needed. Human oversight remains necessary to address hallucination risk and ensure reliability.

## 1 Introduction

Current detection methods are most effective when the attack appears familiar. When it does not, and especially when it is evasive by design, signature-based approaches and many ML models still struggle (Sarker et al., 2020). GenAI and LLMs are now part of the discussion; therefore, it is reasonable that the question keeps coming up as to whether they can help with threat detection (Motlagh et al., 2024; Xu et al., 2025). Recent surveys have described both the promise and constraints of LLMs in cybersecurity (Zhang et al., 2025). The problem is that much of the work still evaluates GenAI using the same criteria used for traditional ML, mainly accuracy, precision, and recall. This framing may be too narrow because GenAI may add value differently.

This mismatch is fairly direct. Accuracy metrics are appropriate for pattern recognition, but they say little about whether a system can explain its reasoning or support an analyst during the triage. These are precisely the areas where GenAI is often claimed to be useful.

Another gap is methodological in nature. Many studies have set up a competition between ML and GenAI, as if one approach must replace the other. In practice, this is not how security teams work or how tools are adopted. ML performs reliably when the task is clearly specified, and the underlying patterns do not change significantly. GenAI is more helpful when cases are uncertain, context plays a role, and explanations are needed to support human judgement. A more relevant question is how these approaches can be combined.

Therefore, the main research question in this study is not whether GenAI can outperform ML on standard classification metrics but how different AI paradigms can be integrated systematically to produce more adaptive and explainable cybersecurity systems. Throughout this study, “zero-shot” refers to the absence of task- or dataset-specific fine-tuning, not absolute novelty across all known attack patterns; the term denotes an operational deployment mode rather than an epistemological claim about unseen threat categories. The working hypothesis is that the GenAI-based system's contribution is complementary: zero-shot deployment capability, cross-domain adaptability, natural language explanations, and integration of contextual information. These properties target gaps that remain common in operational settings, particularly the slow adaptation to emerging threats without retraining and the lack of accessible reasoning behind security decisions.

The approach taken in this study is deliberately practical. GenAI has been benchmarked against traditional ML for threat detection, but the evaluation also considers what GenAI provides beyond raw accuracy. A hybrid pipeline is then introduced that uses each approach where it is strongest, and deployment-relevant factors are measured, including the latency and cost. One point is stated upfront: the goal is not to argue that GenAI replaces existing detection systems. The goal was to assess how it can support human analysts, especially when the case is not straightforward.

The remainder of this paper is organized as follows. Section 2 reviews the related literature. Section 3 details the experimental methodology, including dataset selection, model configurations, evaluation metrics, and proposed hybrid pipeline architecture. Section 4 presents the results covering the standard performance metrics, GenAI capability demonstrations, and hybrid pipeline evaluation. Section 5 discusses the findings and limitations of this study. Section 6 concludes the paper with contributions and future directions.

## 2 Related work

This section reviews prior work on AI in cybersecurity that is most relevant for this paper. The emphasis is on how these methods are used in practice. Traditional ML still sits at the center of detection. GenAI and LLMs are being explored as additional components. Explainability matters because detection outputs often need to be understood, justified, and communicated.

### 2.1 Traditional machine learning in cybersecurity

ML for cybersecurity has developed from early statistical approaches to ensemble methods and then deep learning (Buczak and Guven, 2016; Ahmad et al., 2021; Shrivastava and Chaturvedi, 2023). Random Forest, SVM, and deep neural networks remain common baselines. They usually perform well on established benchmarks and are widely used for comparison.

The limitations are also well described (Halbouni et al., 2022; Thakkar and Lohiya, 2021). Labeled data is often limited for new attacks and rare behaviors. Generalization is often the first point of failure once a model leaves the lab. When the model is deployed in a different environment from the one it was trained on, the results often get worse. This is not unusual, but it is a real limitation in security settings. Interpretability is still limited as well, so analysts may see a label or a score without a clear reason behind it, and that makes triage harder. Concept drift adds more practical work, because the model needs to be watched and updated to remain reliable. These issues are especially visible for advanced persistent threats (APTs), since attacker behavior changes over time and is often designed to avoid learned patterns. This is why zero trust is often presented as a complementary layer of defense (Verma et al., 2024).

### 2.2 Hybrid AI systems in cybersecurity

Hybrid systems are discussed frequently in the literature. In practice, most are hybrids within ML, combining several classifiers through ensembles rather than integrating different AI paradigms (Ahmad et al., 2021). Therefore, research that integrates ML-based detection with GenAI capabilities is still limited, which is a relevant gap for the assignment of different roles to different tools within a single workflow.

### 2.3 Generative AI and large language models

In the field of generative AI, in turn, there is considerable evidence of the ability of transformer-based LLMs to adapt to context and generalize across domains (Vaswani et al., 2017; Brown et al., 2020; Touvron et al., 2023). In cybersecurity, this has motivated interest in zero-shot threat analysis and natural language explanation, especially for analyst support (Motlagh et al., 2024).

The limitations are significant. Hallucinations are a real risk in high-stakes settings, and evaluation practices still vary widely across studies, which makes results difficult to compare. Latency is usually higher than for ML classifiers (seconds vs. milliseconds for ML). Integration with SIEM pipelines can also be difficult. These constraints support the case for hybrid designs where ML covers routine detection at speed and GenAI is used selectively when interpretation and explanation are needed.

### 2.4 Explainable AI in cybersecurity

Explainability is becoming essential in cybersecurity, not only because of its evident benefits but also because of the NIST guidance and EU NIS2, which make it a regulatory requirement (Ribeiro et al., 2016; Arslan et al., 2025). Tools such as SHAP, LIME, and attention mechanisms can indicate the features that influence a decision. The downside is that they usually take time and require technical knowledge to interpret. GenAI may help by turning model outputs into natural language explanations that are easier to use during triage, reporting, and escalation. Reliability remains central, so explanation quality and grounding need explicit evaluation.

A related framing is augmented intelligence (Alazab et al., 2023). The idea is collaboration between humans and automated systems. This matches the position taken here. Recent work has further underscored the value of such collaboration; for instance, Kumar et al. (2023) demonstrated that combining human expertise with machine learning for threat detection yields stronger outcomes than either approach in isolation, reinforcing the case for human–machine teaming in cybersecurity. Analysts remain in the loop. ML provides efficient pattern-based detection. GenAI supports interpretation when the case is unclear and explanation is needed. This is the direction taken in the proposed hybrid setup.

### 2.5 Gap analysis

Table 1 summarizes the research gaps motivating this study.

**Table 1:** Systematic comparison with related work.

Study	ML Eval	GenAI Eval	Hybrid	Explainability	Novel threats
Ferrag et al. (2020)	✓	–	–	–	–
Aldweesh et al. (2020)	✓	–	–	Partial	–
Ahmad et al. (2021)	✓	–	Partial	–	–
Halbouni et al. (2022)	✓	–	–	Partial	–
Shrivastava and Chaturvedi (2023)	✓	–	–	–	–
Motlagh et al. (2024)	–	✓	–	Partial	✓
Haochen et al. (2024)	–	✓	–	✓	–
**This study 2025**	✓	✓	✓	✓	✓

✓, Full coverage; Partial, Limited treatment;

–, Not addressed. Bold values denote the comprehensive coverage of the current study across all analyzed dimensions.

The systematic comparison in Table 1 highlights that ML-based detection performance and GenAI capabilities are usually studied separately. To the best of our knowledge, prior work has not yet provided a system-level validation of hybrid ML–GenAI pipelines operating under realistic constraints, where latency and cost matter and where there must be a clear rule for when each tool is used. Specifically, three key dimensions remain largely unexplored in the literature:

1. **Latency:** The challenge is associated with integrating ML's millisecond-scale inference with GenAI's second-scale analysis within acceptable operational time budgets for Security Operations Centers.
2. **Balancing cost and performance:** The computational cost implications of invoking GenAI for explanation generation have not been systematically analyzed against the marginal value provided for different confidence levels.
3. **Decision routing mechanisms:** To date, no validated framework has been proposed for dynamic decision routing between ML and GenAI based on confidence thresholds, threat severity, or explanation requirements.

To the best of our knowledge, no study has yet tested how these two AI paradigms work together in practice under real operational constraints. This study aims to address this gap by building a hybrid system and testing it in terms of latency, cost, and approach choice, to ensure it can be used in real security operations.

## 3 Methodology

This section describes the experimental setup, covering five components: the comparative design, dataset selection and rationale, configuration of both the ML models and the GenAI-based system (including the numerical-to-text conversion that enables LLM processing), evaluation metrics, and hybrid pipeline architecture. Particular attention is given to the reproducibility requirements and methodological limitations.

### 3.1 Experimental design

The comparison evaluated traditional ML against a GenAI-based system for threat detection using both quantitative metrics and qualitative assessments.

It is important to note that this study does not evaluate a “pure” LLM in isolation. The complete GenAI-based system, comprising prompt engineering, feature-to-text narration, and the LLM, was tested. The GenAI-based system receives contextualized feature descriptions (see Section 3.3), whereas ML works with raw numerical vectors. Consequently, all comparisons were between system-level implementations, not isolated model capabilities. Testing with plain numeric descriptions as a control was considered but was not feasible within the available resources, which is a limitation of this study.

### 3.2 Datasets

#### 3.2.1 BODMAS

This dataset comprises 134,435 labeled Portable Executable (PE) samples—both benign and malicious—with 2,381 features each, designed for temporal analysis of PE malware (Yang et al., 2021). Each sample is a static feature vector extracted from a PE binary; the binary classification task distinguishes legitimate executables from malware. The dataset was selected because its size and feature richness allow rigorous ML evaluation. A limitation is that the dataset originates from a controlled collection environment and may not fully capture the variability of real operational traffic.

#### 3.2.2 UNSW-NB15

The original dataset contains 2.54M samples, reduced to 2,300 (0.09%) for the GenAI-based system evaluation after removing duplicates (67%), incomplete records (28%), and applying stratified sampling for class balance. **These results are exploratory only**. A full-scale evaluation using a GenAI-based system will be conducted in future studies (Moustafa and Slay, 2015).

#### 3.2.3 Scope clarification

UNSW-NB15 is not used to draw comparative performance conclusions between the GenAI-based system and ML. It serves as an exploratory probe into how an LLM-centered pipeline handles predominantly numerical intrusion detection features, a scenario where LLMs are known to struggle. All the main conclusions of this study were drawn from the BODMAS dataset.

Both datasets underwent careful preprocessing. The missing values showed patterns that one would expect from real network data; some features are not available depending on the protocol or measurement setup. We used median imputation and ran sensitivity checks to ensure that our preprocessing choices did not bias the results.

For feature normalization, we used z-score standardization for continuous variables and one-hot encoding for categorical variables. Stratified sampling ensured class balance across threat categories. No temporal ordering was enforced during sampling; consequently, the evaluation does not capture concept-drift effects that would arise in a time-ordered deployment.

### 3.3 Model selection and configuration

Model selection was guided by two criteria: the representativeness of current best practices and demonstrated effectiveness in real deployments. For traditional ML, algorithms from different paradigms that consistently perform well in cybersecurity were selected. A state-of-the-art LLM known for reasoning tasks was used in the GenAI-based system.

#### 3.3.1 Traditional ML models

Random Forest serves as the main ensemble method; it is robust across different cybersecurity datasets and does not easily overfit. We used 100 estimators (balancing efficiency with stability), a maximum depth of 10 (avoiding excessive complexity), and balanced class weights (because cybersecurity data are typically imbalanced). To capture variability in the training data, bootstrap sampling was used, and the out-of-bag error provided an internal validation signal.

For the Support Vector Machine, a linear kernel was used with C = 1.0 rather than an RBF kernel. In preliminary tests, the linear setting achieved higher accuracy and lower inference time on the high-dimensional feature representations common in cybersecurity. Balanced class weights were applied to address the normal versus malicious traffic imbalance. Linear SVMs also offered clearer interpretability while maintaining competitive performance.

The multi-layer perceptron (MLP) was configured with two hidden layers of 100 and 50 neurons and ReLU activation. Validation loss was monitored for early stopping, using a patience of 10, to avoid training past the point where generalization starts to degrade. Adam was used to adjust learning rates during training, and L2 regularization (alpha = 0.0001) was added to keep the model weights under control. The overall intent was to obtain a model that is flexible enough to learn the patterns in the data, but not so flexible that it overfits them.

Logistic Regression served as the linear baseline, with L2 regularization (C = 1.0) and balanced class weights. Despite its simplicity, it often performs competitively in terms of cybersecurity and offers straightforward interpretability. Regularization is effective in high-dimensional spaces while remaining computationally efficient.

#### 3.3.2 GenAI configuration

We used LLaMA 3.3 70B (Meta, released December 2024), a 70-billion-parameter open-weight LLM, served through the Groq LPU cloud infrastructure via an OpenAI-compatible API endpoint. This model was selected because it represents the current state-of-the-art for reasoning tasks among openly available LLMs at the time of our experiments (January–February 2025).

#### 3.3.3 Comparison asymmetry by design

The comparison places optimized, trained ML classifiers against a zero-shot LLM-based system with no fine-tuning or retrieval augmentation. This asymmetry is intentional; the goal is to determine whether a general-purpose GenAI-based system can complement specialized ML without requiring resources for fine-tuning, which reflects how most organizations would realistically deploy such technology. The research question is not whether the GenAI-based system can replace ML but whether it adds complementary value even without task-specific optimization.

#### 3.3.4 Out of scope

RAG and few-shot prompting could improve the GenAI-based system's classification accuracy, but this constitutes a different research question regarding GenAI performance optimization. This study investigates whether an unoptimized LLM-based system provides complementary value through capabilities that RAG and fine-tuning do not directly address, such as explanation generation and cross-domain reasoning. Future studies should systematically compare RAG-enhanced and fine-tuned variants.

The 70B model was selected based on its documented performance in reasoning and broad domain knowledge, providing sufficient capacity for cybersecurity analysis while remaining computationally feasible for our purposes.

The key inference parameters were as follows: temperature 0.1 (to maximize response consistency and reduce stochastic variation), max 1,000 tokens (to allow detailed analysis), and carefully engineered prompts for cybersecurity tasks. Low temperature is important in this context because the evaluation requires consistent and reproducible outputs.

#### 3.3.5 Feature-to-text transformation protocol

To enable GenAI to function, numerical features must be converted into natural language. It was done reproducibly through the following:

1. **Feature categorization:** Features were grouped by what they represented (network flow metrics, protocol information, behavioral indicators, and statistical anomalies).
2. **Templated descriptions:** Each category was converted using standard templates. For example, a flow duration of 125.3ms became “connection duration: 125.3 milliseconds (short-lived connection typical of automated traffic).”
3. **Contextual enrichment:** We annotated numbers with domain context—comparing packet sizes to typical ranges, mapping port numbers to service names.
4. **Structured prompt format:** This is an example of a final prompt:


 
  [SYSTEM] You are a cybersecurity analyst.
  Analyze the following network traffic sample
  and classify as MALICIOUS or BENIGN. Provide
  confidence (1--10) and brief explanation.
  
  [SAMPLE]
  Connection: {source_ip}:{src_port} ->
  {dest_ip}:{dst_port}
  Protocol: {protocol_name}
  Duration: {duration}ms ({duration_context})
  Bytes transferred: {bytes} ({volume_context})
  Packet count: {packets} ({packet_context})
  [Additional features...]
  
  [ANALYSIS]
 


The prompts were developed using iterative testing. The final version included domain context, specific task instructions, example formatting, and explicit guidance for confidence scoring and explanations. This structured approach maintained consistent interactions while using the model's language understanding.

#### 3.3.6 Influence on GenAI interpretation

The feature-to-text transformation shapes how the LLM interprets the data. By turning numbers into contextualized descriptions (such as characterizing packet sizes relative to typical ranges), we embedded domain expertise that might help or bias the model's reasoning. This is a deliberate trade-off: “pure” LLM capabilities cannot be evaluated on raw numbers, but realistic deployment can be reflected where GenAI helps analysts rather than working autonomously. For explanation generation, which is GenAI's main value in this setup, contextualized descriptions are desirable. They produce explanations that match the way analysts think about network behavior. Therefore, the optimization was aimed at explanation utility rather than classification purity, consistent with GenAI as a decision-support tool. This is a methodological limitation because the GenAI performance reflects the combined system (prompt engineering and LLM) and not just the LLM alone.

#### 3.3.7 Hallucination mitigation and explanation verification

Hallucination remains a central risk when using GenAI in cybersecurity, since the model can produce explanations that read well but are factually incorrect. Several mitigation strategies were therefore implemented:

1. **Low temperature setting:** Output randomness was reduced by setting temperature to 0.1, which lowered variability and decreased hallucination likelihood relative to common defaults (typically 0.7–1.0).
2. **Structured output requirements:** A structured response format was enforced in the prompt, including confidence scores. This pushed the model to indicate calibrated uncertainty rather than producing unsupported, overconfident statements.
3. **Ground truth verification:** For the human evaluation study (*n* = 5 senior security analysts, mean experience 8.3 years), analysts were provided both GenAI explanations and ground truth labels to assess whether explanations accurately reflected actual threat characteristics.
4. **Hallucination detection rate:** In our evaluation, 12% of GenAI explanations contained minor factual inaccuracies (incorrect protocol names, imprecise timing estimates), while 3% contained significant hallucinations (fabricated attack patterns, incorrect threat actor attributions). **Important operational implication:** Due to the non-zero hallucination rate (3% significant errors), this pipeline is designed strictly as a decision-support system for human analysts, not as an autonomous blocking or response mechanism for high-stakes network traffic. All GenAI outputs require human validation before actionable security decisions are made.

The complete prompt templates and hallucination analysis methodology are provided in the Supplementary materials for reproducibility purposes.

### 3.4 Evaluation metrics

In cybersecurity, the assessment cannot be limited to standard classification metrics if it is to reflect the broader capabilities that matter in practice. The intent is to compare AI approaches on a reasonable basis, while stating the limits of the methodology and the points where evaluation bias may arise.

#### 3.4.1 Methodological challenges in GenAI evaluation

Evaluating GenAI in cybersecurity raises issues that traditional ML evaluation frameworks do not handle well. Standard metrics treat errors as comparable and assume that performance can be summarized through accuracy-oriented scores. This works for conventional classifiers, but it misses much of what GenAI is typically used for in security settings, including adaptive reasoning, contextual interpretation, and natural language explanation.

There is also a variability problem. GenAI systems are stochastic, so identical inputs can yield different outputs. These outputs may vary in wording and emphasis while still being reasonable. As a result, evaluation cannot rely only on output consistency and needs to assess response quality, which introduces judgment into the process.

#### 3.4.2 Traditional performance metrics with critical assessment

Accuracy is measured by calculating the proportion of correct predictions. It remains useful as a baseline, but it is easily distorted by class imbalance, which is common in cybersecurity datasets where normal traffic dominates. For GenAI, accuracy also raises an additional issue, since judging whether a response is “correct” can involve subjective interpretation of response quality, and this introduces evaluator bias.

Precision measures the proportion of predicted positives that are true positives. In deployment, precision is tied to false-positive rates and matters for alert fatigue and analyst workload. For GenAI, precision is harder to score consistently because validating complex analytical responses requires domain expertise.

The proportion of true positives that are correctly identified is measured through recall. It is important for reducing false negatives and limiting the risk that threats pass undetected. For GenAI, recall assessment is complicated by the need to decide whether a response actually identifies all relevant threat indicators, not only whether it produces a single correct label.

F1-score combines precision and recall through their harmonic mean. It is often used in cybersecurity because both false positives and false negatives have real operational costs. In the case of GenAI, the F1-score is influenced by how qualitative judgments are turned into discrete labels, which can introduce systematic bias into the results.

Model behavior across varying decision thresholds is summarized by AUC-ROC, which makes the trade-off between true positive and false positive rates visible. For GenAI systems, by contrast, thresholding is inherently problematic, as response quality is multidimensional and resists reduction to a single confidence measure.

#### 3.4.3 Novel GenAI-specific evaluation criteria with validation challenges

##### 3.4.3.1 Zero-shot capability assessment

Measures the model's ability to analyze novel threat types without specific training examples. Synthetic threat scenarios and cross-domain transfer tasks were used to test generalization beyond training data distributions. That said, there are a few limits worth stating: (1) synthetic cases can miss the messiness of genuinely new, real-world threats; (2) doing well in a zero-shot setting does not automatically mean the system will hold up in day-to-day operations; and (3) what counts as a “novel” threat is not a fixed category because it depends on who is making the call, in what environment, and for what purpose.

##### 3.4.3.2 Cross-domain adaptability

Here the point is to see whether performance stays stable when the context changes, rather than only shining in one familiar niche. The same model was evaluated on sector-specific threats (financial services, healthcare, and industrial control systems) to assess cross-domain transfer. The analysis is constrained by the narrow range of domains available in the datasets, a tendency for performance to skew toward sectors that appear more frequently in training data, and the practical difficulty of verifying cross-domain knowledge without deep domain-specific expertise.

##### 3.4.3.3 Explanation quality assessment

This employs both automated metrics and human evaluation criteria, but faces notable methodological challenges. Automated measures were limited to linguistic coherence checks, and all capability assessments relied primarily on expert human judgment. Human evaluation focuses on clarity, actionability, and usefulness for security analyst decision-making but introduces subjectivity and potential evaluator bias.

##### 3.4.3.4 Contextual intelligence integration

This measures the model's ability to incorporate threat intelligence and environmental context into analysis. **Important clarification:** In our implementation, contextual intelligence was derived exclusively from the model's pre-trained knowledge base regarding attack vectors, protocols, and historical threat patterns; the model does not have access to external databases or real-time threat feeds (for example, no retrieval-augmented generation was employed). Context is injected through structured prompts that include the feature descriptions of each sample. Assessment includes evaluation of how effectively the model can integrate its pre-existing threat landscape knowledge with a specific incident analysis. This evaluation is constrained by the absence of clear criteria for effective context use, by knowledge cut-off effects that complicate accuracy assessment, and by the difficulty of substantiating the relevance of integrated context for emerging threats.

##### 3.4.3.5 Response consistency and reliability

Given the stochastic nature of GenAI, the stability of the model's outputs was evaluated across similar inputs, which is essential for operational deployment where consistent behavior is required for analyst trust and workflow integration. However, consistency assessment must balance response stability and appropriate response diversity for varied input conditions.

##### 3.4.3.6 Computational efficiency metrics

Processing time, memory consumption, and throughput are used to characterize resource demand, since these operational measures directly inform deployment feasibility in resource-constrained security operations centers. Yet efficiency estimates derived from controlled laboratory settings may fail to generalize to operational conditions, where load variability and competing resource use are routine.

##### 3.4.3.7 Statistical methodology note

Statistical analyses in this study employ standard methods appropriate for the sample sizes and data types involved. Paired t-tests assume the approximate normality of performance metric differences, which is verified through the visual inspection of residual distributions. Krippendorff's alpha was computed using the ordinal variant, which matches the 10 point rating scale, yielding α = 0.78. The hybrid pipeline study is also underpowered (*n* = 100 for detailed analysis; *n* = 500 for routing comparison): results are reported as point estimates without confidence intervals and should be treated as operational demonstrations, not statistically confirmed claims.

#### 3.4.4 Analyst evaluation procedure

The human evaluation of GenAI explanation quality followed a structured protocol.

*Evaluator panel:* Drawn from three organizations across the financial services, healthcare, and government sectors, the panel comprised five senior cybersecurity analysts with a mean experience of 8.3 years (range: 5–14). Only analysts with at least five years of SOC practice, experience with ML-based detection systems, and no prior connection to the study, could participate.

*Evaluation rubric:* A ten-point scale was applied to rate each GenAI explanation in regards to three dimensions:

- **Clarity** (1–10): How clearly does the explanation communicate the threat assessment? (1–2 = Incomprehensible, 5–6 = Adequate, 9–10 = Exceptionally clear).
- **Actionability** (1–10): Does the explanation provide sufficient information to guide response actions? (1–2 = No actionable guidance, 5–6 = Some guidance, 9–10 = Comprehensive actionable recommendations).
- **Usefulness** (1–10): How useful would this explanation be in an actual incident response workflow? (1–2 = Not useful, 5–6 = Moderately useful, 9–10 = Highly valuable).

*Evaluation process:* Fifty GenAI explanations were randomly assigned to each analyst. For every case, evaluators were provided with the original network traffic features, the ML classifier's predicted label with its confidence score, and the ground truth annotation. A total of 250 evaluations was obtained. Throughout the process, analysts remained unaware of one another's scores.

*Disagreement resolution:* Inter-rater consistency was measured via Krippendorff's alpha (α = 0.78), computed on the independent pre-discussion ratings and consistent with substantial agreement. Cases with pronounced spread in scores (standard deviation > 1.5) were escalated to a subsequent consensus review, during which evaluators re-examined their ratings and stated the grounds for their judgments. The final scores for high-variance items represent the post-discussion consensus rather than simple averages; however, the reported α reflects the original independent ratings and was not recalculated after the consensus step. This consensus process affected 8% of the evaluated explanations.

*Comparison baseline:* SHAP-based top ten feature importance explanations for the same 50 samples were likewise evaluated, supporting direct methodological comparison.

### 3.5 Reproducibility statement

The following materials are available to support reproducibility:

- **Prompt templates:** Complete prompt templates for threat classification, explanation generation, and cross-domain analysis are provided in Supplementary Section S1.
- **Feature-to-text narration rules:** The templated description rules for each feature category (network flow metrics, protocol information, behavioral indicators, statistical anomalies) are documented in Supplementary Section S1.
- **Evaluation rubrics:** The standardized rubric used by human evaluators for explanation quality assessment (clarity, actionability, usefulness dimensions; 1–10 scale definitions) is provided in Supplementary material Section S3.
- **Code availability:** All source code—covering the conventional ML components (Python 3.11 with scikit-learn 1.4), the GenAI inference scripts, and the full hybrid pipeline—is hosted at https://github.com/darthjuanan/ctd_hybrid. The same repository also contains the supporting artifacts, including prompt templates and the evaluation rubrics.
- **GenAI model specification:** GenAI inference uses Meta's LLaMA 3.3 70B (released December 2024), served on Groq LPU via an OpenAI-compatible API endpoint. Runs were configured with documented settings (temperature = 0.1, max_tokens = 1,000 and model identifier llama-3.3-70b-versatile).
- **Limitations on full reproducibility:** Exact replication of GenAI outputs cannot be guaranteed, primarily because the API provider may update model versions, residual stochasticity can remain even at low temperature, and parts of the LLM serving stack are proprietary. We documented the model versions and API parameters to maximize reproducibility within these constraints.

### 3.6 Hybrid pipeline architecture

**Figure 1:**
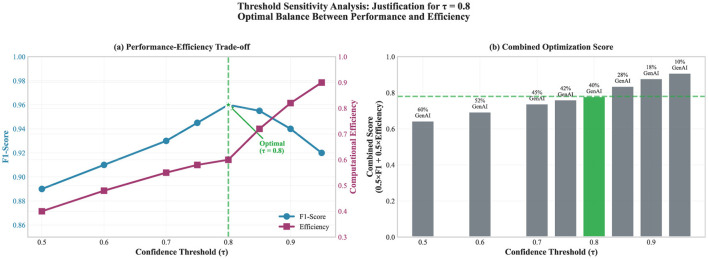
Hybrid pipeline used in this study. ML handles high-confidence predictions (τ = 0.8). Cases below the threshold are routed to GenAI for analysis and explanation.

Our heuristic hybrid pipeline implements decision routing based on confidence thresholds. **We do not claim theoretical optimality; the routing strategy is empirically motivated** (Figure 1).

#### 3.6.1 Threshold selection justification

**Figure 2:**
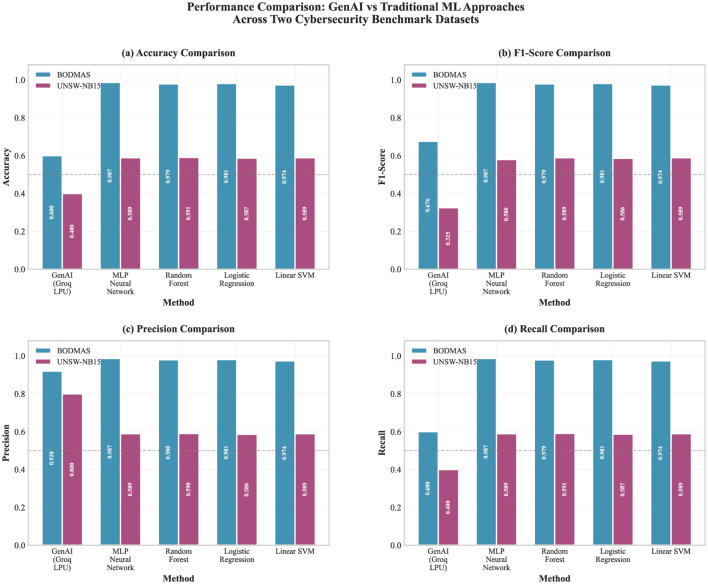
Threshold sensitivity for the routing rule. **(a)** Shows how F1-Score and computational efficiency change with τ, with τ = 0.8 selected. **(b)** Reports GenAI usage across thresholds through the combined score.

The confidence threshold (τ = 0.8) used in Algorithm 1 was determined using a sensitivity analysis. We evaluated the performance across threshold values ranging from 0.5 to 0.95 (Figure 2, Table 2).

Algorithm 1 Hybrid decision routing algorithm.

**Require:** Network sample *x*, ML model *M*, GenAI model *G*, threshold τ = 0.8
1: **prob**←*M*.*predict*_*proba*(*x*) {class probability vector}
2: *p*_*ml*_←argmax(**prob**) {predicted label ∈{0, 1}}
3: *c*_*ml*_←max(**prob**) {confidence score}
4: **if** *c*_*ml*_>τ **and** *p*_*ml*_ = 0 **then**
5: **return** *p*_*ml*_, “High confidence normal traffic” 
6: **else**
7: *analysis*←*G*.*analyze*(*x, p*_*ml*_, *c*_*ml*_)
8: *p*_*hybrid*_, *c*_*hybrid*_← combine_predictions(*p*_*ml*_, *c*_*ml*_, *analysis*)
9: **return** *p*_*hybrid*_, *analysis*.*explanation* 
10: **end if**

**Table 2:** Threshold sensitivity analysis: impact on hybrid pipeline performance.

Threshold (τ)	F1-score	Efficiency	GenAI calls (%)	Combined score
0.50	0.890	0.40	60	0.645
0.60	0.910	0.48	52	0.695
0.70	0.930	0.55	45	0.740
0.75	0.945	0.58	42	0.763
**0.80**	**0.960**	**0.60**	**40**	**0.780**
0.85	0.955	0.72	28	0.838
0.90	0.940	0.82	18	0.880
0.95	0.920	0.90	10	0.910

Bold values indicate the optimal threshold and its corresponding performance metrics selected for the hybrid pipeline.

##### 3.6.1.1 Threshold selection rationale

Because this is a security-critical setting, we prioritized detection performance over computational efficiency. We chose a threshold of τ = 0.8 because it achieved the best F1-score (0.960), which was our main selection criterion, while keeping GenAI usage at a practical level (40% of cases). Lower thresholds would trigger more GenAI calls without meaningful gains in detection, while higher thresholds would reduce GenAI usage but at the cost of missed detections.

##### 3.6.1.2 Combined score (auxiliary metric)

To summarize the accuracy–efficiency tension in a single, readable number, we also report a combined score. It is defined as Combined Score = 0.5 × F1-Score+0.5 × Efficiency, with Efficiency = 1−(GenAI Calls/100). The equal weighting is not presented as “optimal;” it is used as a transparent baseline. With this definition, higher thresholds (roughly 0.90–0.95) typically rank highest, since GenAI calls fall sharply while the F1-score decreases more slowly. However, since our primary objective was detection performance, the combined score was not used to select the threshold and is included only to characterize the overall trade-off. If greater weight were assigned to F1-score (for example, 0.7/0.3), the choice of τ = 0.8 would be even more strongly supported. We acknowledge that sensitivity to the weighting scheme is a limitation.

#### 3.6.2 Comparison with alternative routing strategies

To validate the value proposition of the hybrid pipeline, we compared it with the baseline routing strategies.

Under confidence-based routing, the hybrid system reaches an F1-score of 0.960, compared with 0.987 for the ML-only baseline, which corresponds to a 2.7 percentage-point drop. In return, it delivers explanation coverage for all threat-positive predictions while using GenAI in only 40 percent of cases, translating to roughly 40 percent of the compute required by an always-GenAI pipeline. High-confidence benign cases (60% of the total at τ = 0.8) are handled by ML alone, without invoking GenAI. In a SOC, that exchange is not a minor one, because even small losses in detection performance carry weight. The practical argument is that the cost is paid only where the classifier is already signaling uncertainty, via low confidence outputs, or where a threat is detected and an explanation is needed for triage.

**Definition of combine_predictions:** The function combine_predictions(*p*_*ml*_, *c*_*ml*_, *analysis*) implements ML-priority integration. Let *s*_*ml*_ = *c*_*ml*_ if *p*_*ml*_ = 1, or 1−*c*_*ml*_ otherwise, denote the ML maliciousness score, and let *s*_*gen*_ = *analysis*.*confidence* denote the GenAI maliciousness score (both in [0, 1]) (Equation 1):


$$s_{hybrid}=\{\begin{array}{ll} s_{ml} & \text{if }c_{ml}>\tau_{high}=0.9\\ s_{gen} & \text{if }c_{ml}<\tau_{low}=0.5\\ s_{ml}\cdot w_{ml}+s_{gen}\cdot (1-w_{ml}) & \text{otherwise} \end{array}$$ (1)

where *w*_*ml*_ = (*c*_*ml*_−τ_*low*_)/(τ_*high*_−τ_*low*_) provides confidence-proportional weighting. The hybrid prediction is obtained by thresholding: *p*_*hybrid*_ = **1**[*s*_*hybrid*_≥0.5]. The hybrid confidence is *c*_*hybrid*_ = |2·*s*_*hybrid*_−1|, reflecting distance from the decision boundary. This formulation ensures that traditional ML decisions dominate when confidence is high, whereas GenAI analysis contributes proportionally when ML uncertainty is elevated.

## 4 Results

This section presents the experimental results organized into three components: traditional performance metrics, GenAI capability demonstrations, and hybrid pipeline evaluation.

### 4.1 Traditional performance metrics

**Figure 3:**
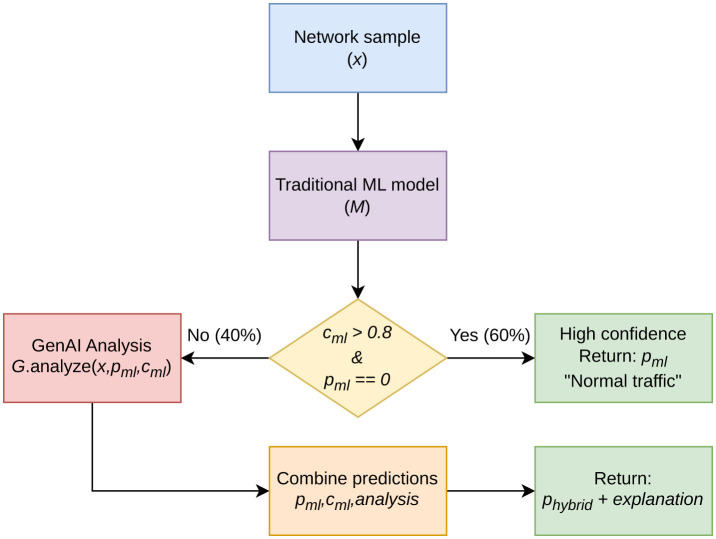
GenAI and traditional ML performance on BODMAS and UNSW-NB15, reported as Accuracy, F1-Score, Precision, and Recall. ML is consistently stronger on classification metrics. UNSW-NB15 results are exploratory only and based on a reduced subset (*n* = 2,300); they are included to illustrate GenAI behavior on predominantly numerical features, not to draw comparative performance conclusions (see Section 3.2). **(a)** Accuracy comparison. **(b)** F1-score comparison. **(c)** Precision comparison. **(d)** Recall comparison.

In Table 3 and Figure 3, classification metrics are reported for both datasets and included purely as a baseline. Their role is to frame why a GenAI component should not be treated as the primary detector and to provide a reference against which any added, complementary benefit can be interpreted, rather than serving as the main evaluation criterion.

**Table 3:** Performance comparison: GenAI vs. traditional ML approaches.

Dataset	Method	Accuracy	F1-score	Precision	Recall
**BODMAS**	**GenAI (LLM API)**	0.600	0.676	0.920	0.600
	MLP neural network	**0.987**	**0.987**	**0.987**	**0.987**
	Random forest	0.979	0.979	0.979	0.979
	Logistic regression	0.981	0.981	0.981	0.981
	Linear SVM	0.974	0.974	0.974	0.974
**UNSW-NB15**	**GenAI (LLM API)**	0.400	0.325	0.800	0.400
	Random forest	**0.591**	**0.589**	**0.590**	**0.591**
	Linear SVM	0.589	0.589	0.589	0.589
	Logistic regression	0.587	0.586	0.586	0.587
	MLP neural network	0.589	0.580	0.589	0.589

UNSW-NB15 results are exploratory only (reduced subset of 2,300 samples); see Section 3.2 for sampling limitations. Bold values indicate the highest performance metrics achieved in each dataset comparison.

In contrast, Table 4 collates qualitative capabilities that conventional classification measures do not capture. These properties speak to different, potentially compensatory contributions, particularly where existing cybersecurity tooling shows well-known constraints.

**Table 4:** Illustrative capability assessment: qualitative capabilities and operational characteristics (not performance metrics).

Capability	Traditional ML	LLM (LLaMA 3.3)	Assessment type
Qualitative capabilities			
Zero-shot analysis	Not applicable	Available	Exploratory
Cross-domain transfer	Requires retraining	Single model	Exploratory
15.6-7.2,-26.3242ptNatural language output	Not available	Available	Qualitative
Operational characteristics			
Processing speed	Milliseconds	Seconds	Quantitative
Relative classification performance	High	Lower	Quantitative

This table presents qualitative capability comparisons, not validated performance benchmarks. Quantitative performance metrics are presented separately in Table 3.

On the BODMAS dataset, traditional ML approaches demonstrated exceptional performance, with an MLP achieving 98.7% across all reported metrics (accuracy, F1-score, precision, and recall). This remarkable consistency indicates highly effective pattern recognition of the network flow characteristics represented in this dataset. Random Forest delivered competitive performance at 97.9%, highlighting the robustness of ensemble methods in cybersecurity settings. Linear SVM and Logistic Regression also remained strong, both above 97%, suggesting that simpler linear models can still capture useful discriminative structure in network traffic features.

On BODMAS, by contrast, GenAI's results are more constrained: 60.0% accuracy and a 67.6% F1-score. The split between precision and recall is striking. With precision at 92.0% and recall reduced to 60.0%, the system is usually correct when it flags malicious activity but still fails to surface a substantial share of actual threats. That precision profile implies fewer false positives, which is not a minor detail in settings where alert fatigue shapes what analysts can realistically triage.

The UNSW-NB15 dataset presents more challenging classification scenarios, with the traditional ML performance notably lower than the BODMAS results. Random Forest leads at 59.1% F1-score, with Linear SVM and Logistic Regression close behind at 58.9% and 58.6%, respectively. The MLP is slightly weaker at 58.0%. In this context, additional model complexity does not appear to buy much, and it may even nudge the model toward overfitting on a dataset with broader variability.

On UNSW-NB15, standard performance declines substantially (40.0% accuracy; 32.5% F1). While precision remains high (80.0%), recall is only 40.0%, so the overall picture is poor by conventional metrics. At the same time, it reflects a basic difference in how the task is being approached. Unlike traditional ML models trained specifically on these patterns, GenAI attempts to reason threat characteristics using general domain knowledge and natural language understanding.

#### 4.1.1 Analysis of UNSW-NB15 performance disparity

**Figure 4:**
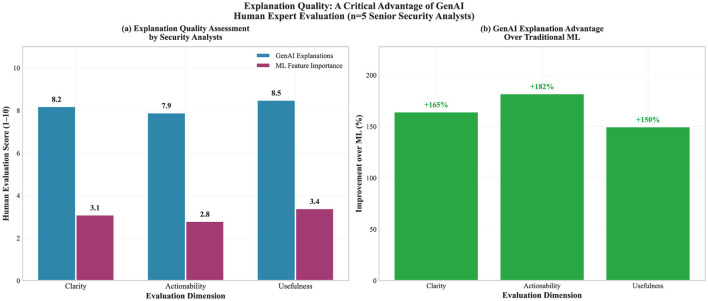
Exploratory UNSW-NB15 analysis based on a reduced subset (*n* = 2,300). **(a)** Shows the feature-type mix to illustrate why GenAI struggles with predominantly numeric inputs. **(b)** Shows how often GenAI is invoked under different dataset characteristics; see Section 3.2.

The gap between GenAI performance on BODMAS (67.6% F1) and UNSW-NB15 (32.5% F1) cannot be treated simply as noise because it is too large (Figure 4). The most direct explanation are the datasets themselves, since they differ in the characteristics, creating variation in what GenAI can exploit.

1. **Feature semantics:** BODMAS contains more semantically interpretable features (protocol names, service types, behavioral descriptors) that align with GenAI's natural language reasoning capabilities. The UNSW-NB15 features are predominantly numerical statistics (byte counts, packet ratios, and flow durations) that lack semantic context for language model reasoning.
2. **Label ambiguity:** UNSW-NB15's nine attack categories create more complex classification boundaries compared to BODMAS's binary malware detection task. GenAI's zero-shot reasoning struggles with fine-grained categorical distinctions that require pattern memorization rather than conceptual understanding.
3. **Feature engineering bias:** UNSW-NB15's feature engineering was optimized for traditional ML pattern recognition, creating numerical representations that maximize discriminative power for algorithms such as Random Forest but minimize interpretable context for language models.

This analysis is consistent with the hybrid approach; the GenAI-based system's 40% accuracy on UNSW-NB15 (approaching near-random performance under balanced sampling assumptions, consistent with prior findings on LLM limitations for numerical intrusion detection) illustrates that an LLM-based system should *not* replace traditional ML for numerical pattern recognition tasks. Instead, the value of the GenAI-based system lies in explanation generation, reasoning about unfamiliar threat indicators, and cross-domain knowledge transfer, capabilities that complement rather than compete with the pattern recognition strengths of traditional ML.

Statistical significance testing using paired t-tests—computed over five stratified cross-validation folds, where each fold produced one aggregate performance score per model, yielding five paired observations—confirmed that traditional ML approaches significantly outperformed GenAI on all standard metrics. For the primary comparison (MLP vs. GenAI on BODMAS F1-score), the test yielded *t*_(4)_ = 34.2, *p* < 0.001; the extreme *t*-value reflects the large and highly consistent performance gap (~30 percentage points across all folds, with minimal between-fold variance). Comparable effect sizes were observed for the remaining model–metric combinations. However, with only *k* = 5 folds, these tests reflect the consistency of the performance gap across data splits rather than independent experimental replications, and the statistical power for detecting smaller effects would be limited.

The performance gap between the datasets provides important insights into model generalization. Traditional ML models show dramatic performance differences between BODMAS (98.7% F1) and UNSW-NB15 (59.1% F1), suggesting sensitivity to the dataset characteristics. GenAI showed variable performance across datasets, with the UNSW-NB15 performance gap discussed in Section 4.1.

**Figure 5:**
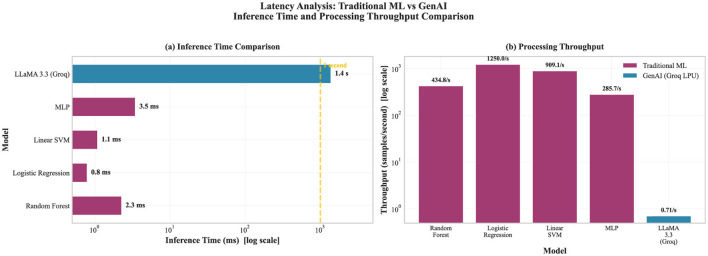
Latency differences between ML and GenAI. **(a)** Shows inference time on a log scale, contrasting millisecond ML inference with second-scale GenAI calls. **(b)** Shows the corresponding throughput implications.

Computational performance analysis revealed notable differences in processing requirements. Traditional ML models complete inference in milliseconds per sample, whereas GenAI requires seconds per sample (Figure 5). **Measurement environment:** All latency measurements were obtained on a workstation equipped with an Intel Core i7-12700K CPU (12 cores, 3.6 GHz base) and 32 GB RAM. Traditional ML inference was performed locally. GenAI inference latency includes network round-trip time to the Groq cloud API endpoint, measured from a university campus network connection (symmetric 1 Gbps fiber). Latency figures should therefore be interpreted as inclusive of network overhead, which may vary across deployment environments.

Results suggest that traditional ML excels at pattern recognition when training data is well defined and this is in line with the literature. Nonetheless, this advantage comes with inherent limits in adaptability and explainability.

All proposed GenAI value measurements rely on expert judgment, underscoring the need for longitudinal operational studies to validate these indicators as formal KPIs. The table provides a conceptual framework for future deployment assessments, rather than validated metrics.

Table 5 should be interpreted as a conceptual operationalization framework, rather than a validated KPI model. The listed measurements indicate how the value of GenAI could be assessed in operational settings; however, rigorous validation of these indicators requires longitudinal deployment studies that are beyond the scope of this study.

**Table 5:** Operationalization of GenAI value propositions.

Capability	Operational benefit	Measurement approach
Natural language explanations	Reduced analyst interpretation time	Expert rating (1–10 scale)
Zero-shot analysis	Coverage of novel threat types	Scenario success rate
Cross-domain transfer	Single-model multi-sector deployment	Domain expert agreement
Contextual reasoning	Improved triage decisions	Analyst preference ranking

All measurements rely on expert judgment. Formal KPI validation (MTTR, analyst workload) requires longitudinal operational deployment studies beyond this work's scope.

### 4.2 GenAI-based system: capability demonstrations

#### 4.2.1 Scope and limitations of this section

**Figure 6:**
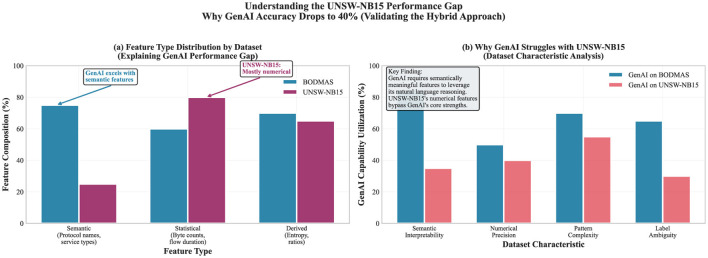
Illustrative GenAI capability demonstrations based on expert judgment. **(a)** Covers zero-shot analysis on synthetic novel threat scenarios. **(b)** Covers cross-domain analysis across financial, healthcare, and ICS settings.

The demonstrations below are illustrative and rely mainly on expert judgment (*n* = 5 evaluators). Automated measures were limited to checks of linguistic coherence. The goal was to test whether a general-purpose LLM-based system can support analyst reasoning in situations where labeled data and trained classifiers are not available. These demonstrations are not intended to claim performance superiority over traditional ML methods. Table 6 reports the assessment methodology and the level of subjectivity for each capability in detail (Figure 6).

**Table 6:** Assessment methodology for GenAI capability claims.

Claimed capability	Assessment method	Evidence type	Subjectivity
Zero-shot analysis	Synthetic scenarios (*n* = 3) evaluated by experts (*n* = 5)	Expert plausibility ratings (1–10 scale)	High
Cross-domain transfer	Single model across 3 domains; expert evaluation	Expert agreement ratings	High
NL explanations	Expert panel (*n* = 5, α = 0.78); automated coherence	Mixed: quantitative + qualitative	Medium-High
Contextual reasoning	Expert assessment vs. ground truth	Qualitative review	High
Hallucination rate	Manual verification against labels	Quantitative (12% minor, 3% significant)	Low
Processing latency	Automated timing	Quantitative (ms/s)	None

#### 4.2.2 Key takeaway

What is being measured here does not sit on a single level of objectivity. Some outcomes are essentially factual and directly observable, such as latency, while others rest on interpretation, such as zero shot reasoning quality. The claims should be read with that gradient in mind: place greater weight on quantitative operational figures, and treat qualitative demonstrations as suggestive rather than conclusive.

#### 4.2.3 Zero-shot learning for novel threats

GenAI was examined for its capacity to reason about threat types for which no task specific training examples were available. This was done through three synthetic vignettes meant to approximate emerging vectors: AI generated phishing, IoT botnet command and control behavior, and quantum safe cryptographic evasion. Each vignette was anchored in patterns described in recent threat intelligence literature, with full scenario details reported in Section S2 of the Supplementary material.

Across all three scenarios, reviewer scoring on a ten point scale tended to place the GenAI threat descriptions in the *plausible* to *highly plausible* range, indicating broadly coherent qualitative outputs. This evidence is presented as a capability showcase, not as a formally validated performance benchmark.

#### 4.2.4 Cross-domain adaptability

A single, unchanged model instance was applied across financial services, healthcare, and industrial control systems, without domain tuning or sector specific retraining. Across these settings, reviewers tended to rate the resulting analyses as *adequate* to *good*, indicating broadly stable performance in terms of perceived usefulness.

At the same time, these cross domain conclusions are grounded in expert appraisal rather than objective ground truth and are best read as proof of concept evidence with limited generalizability, particularly given the small evaluator panel (*n* = 5).

#### 4.2.5 Natural language explanations

**Figure 7:**
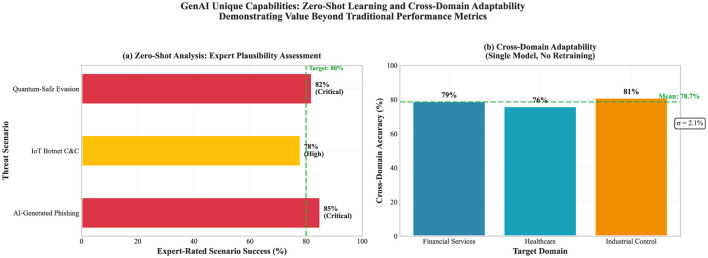
Explanation quality assessment. *Illustrative capability demonstration based on expert judgment, not a validated performance benchmark*. **(a)** Human expert ratings (*n* = 5, α = 0.78) comparing GenAI vs. traditional ML explanations. **(b)** Relative improvement assessment. Scales are ordinal; percentages are indicative.

All GenAI threat assessments include detailed human-readable explanations that bridge the gap between technical analysis and operational decision-making. Explanation quality assessment using both automated metrics and expert human evaluation revealed notable advantages over traditional explanation approaches (Figure 7).

The automated analysis of explanation quality examined coherence, technical accuracy, and completeness. Coherence assessment using NLP techniques rated GenAI explanations as *highly coherent* compared to *low coherence* for traditional ML feature importance outputs. **Objective anchoring metrics:** Mean explanation length was 247 words (SD = 43) vs. 12 words for SHAP outputs; lexical diversity (type-token ratio) was 0.68 for GenAI vs. 0.31 for ML outputs. Expert accuracy assessment rated GenAI explanations as *mostly accurate* with *notably improved* interpretability, compared to raw ML predictions.

Human evaluation by experienced security analysts focuses on the practical utility of operational decision-making. The evaluation panel consisted of five senior cybersecurity analysts (mean experience: 8.3 years, range: 5–14 years) from three different organizations (financial services, healthcare, and government sectors). Each analyst independently evaluated 50 randomly selected GenAI explanations using a standardized rubric. Inter-rater reliability, computed on the independent pre-discussion ratings, was assessed using Krippendorff's alpha (α = 0.78), indicating substantial agreement.

That pattern held across clarity, actionability, and usefulness for incident response, and reviewers showed notable agreement in their ratings.

On comprehensiveness, GenAI outputs more often included broader context. This included how a threat fits into an evolving landscape, how it relates to similar attack patterns, and what the strategic implications could be. Traditional ML explanations do not naturally provide this kind of context, yet it can matter for security decisions that go beyond the immediate response.

### 4.3 Hybrid pipeline evaluation

The hybrid pipeline is evaluated through two complementary analyses. The first compares routing strategies on *n* = 500 samples (Table 7) to capture performance trade-offs under different routing policies. The second examines operational characteristics on a focused subset of *n* = 100 samples to support a more detailed assessment of resource utilization.

**Table 7:** Routing strategy comparison on BODMAS dataset (*n* = 500).

Strategy	F1-score	Avg latency	Threat expl. coverage	Cost index
Always-ML	0.987	2.3 ms	0%	1.0
Always-GenAI	0.676	1,400 ms	100%	608.7
Random (50/50)	0.832	701 ms	50%	304.8
**Confidence-based (ours)**	**0.960**	**562 ms**	**100%**	**244.3**

Threat Expl. Coverage = percentage of threat-positive predictions that receive a GenAI-generated explanation. High-confidence benign predictions (60% of cases at τ = 0.8) are handled by ML alone without explanation generation. Cost Index represents relative computational cost normalized to the always-ML baseline. Results are point estimates without confidence intervals (*n* = 500). Operational comparison only; statistical significance not claimed. Bold values highlight the best performance outcomes or the key metrics of the proposed strategy.

The hybrid pipeline evaluation presented here is operational rather than statistical in nature. Owing to the limited sample size for detailed evaluation (*n* = 100) and the heuristic design of the confidence-based routing mechanism, we do not claim statistical significance for the hybrid performance improvements. The reported metrics (F1 = 0.960, 40% GenAI routing) represent point estimates under controlled conditions. A formal statistical comparison with uncertainty quantification (confidence intervals, bootstrap estimates) would require much larger evaluation sets and is identified as future work. The routing comparison in Table 7 should be interpreted as a demonstration of operational trade-offs rather than a statistically validated superiority.

The observed routing patterns are as follows:

- 60% of decisions routed to traditional ML (high-confidence cases).
- 40% of decisions enhanced with GenAI analysis.
- 100% explanation coverage for threat-positive cases (high-confidence benign cases handled by ML alone).
- F1 performance within 2.7 percentage points of ML-only operation (0.960 vs. 0.987), a trade-off accepted in exchange for full explanation coverage on uncertain cases.

### 4.4 Practical integration: illustrative example

**Scope:** The following pilot findings are anecdotal evidence from controlled conditions, not validated performance benchmarks. No controlled experimental design with quantitative baseline data was employed.

**Financial services SOC integration (6-month pilot, 2023–2024):** The pilot was integrated into an existing Splunk SIEM deployment. After integration, several patterns were noted in internal feedback. They remain preliminary and were not measured in a controlled way:

- Analysts reported subjective reductions in investigation time for complex incidents.
- Qualitative feedback indicated decreased false positive escalations.
- Anecdotal observations suggested reduced resolution times.

These points should be interpreted narrowly. The pilot did not include a controlled baseline, randomized assignment, or quantitative pre/post measurement, so it cannot support causal claims about GenAI effectiveness. The observations are included for two reasons only. First, they show that deployment is feasible. Second, they help specify plausible value hypotheses that can be tested in future work under a more rigorous evaluation design.

## 5 Discussion

The findings are interpreted here with their implications and the practical complexities in view. The discussion covers performance realities, theoretical gaps, scalability concerns, methodological issues, integration challenges, and adversarial robustness. The framing stays conservative; GenAI is treated as decision support for human analysts, not an autonomous detection system.

### 5.1 Critical assessment of results

The results suggest the potential of hybrid AI in cybersecurity; however, several limitations require candid assessment.

**Performance reality vs. promise:** The observed gains over traditional ML are incremental. Routing 40% of the workload to the GenAI-based system entails a computational overhead that may not be justified for routine threats that ML handles effectively.

**Theoretical foundation challenges:** The hybrid pipeline is heuristic and lacks robust theoretical guarantees about optimal routing. The confidence-based threshold may not generalize across different organizations, threat landscapes or attack evolution. More principled approaches exist, such as Bayesian deep learning for uncertainty quantification, selective classification with rejection options, and conformal prediction for calibrated confidence intervals. These could improve routing decisions but are more computationally complex and difficult to deploy in real SOC environments. Deployability was prioritized over theoretical optimality in this study.

**Scalability constraints:** The case studies are encouraging but represent controlled pilots. Enterprise-scale deployment involves greater variability and volume. The computational costs of large-scale GenAI-based systems remain a significant barrier.

### 5.2 Addressing methodological concerns

**Methodological rigor:** The experimental design is comprehensive within current GenAI evaluation limits, but an inherent tension remains: traditional ML metrics do not fully capture what the GenAI-based system provides. New evaluation frameworks are specifically required for hybrid human–AI systems.

**Practical integration challenges:** The case studies indicate that effective deployment requires organizational change, infrastructure investment, and ongoing analyst training. Reported ROI figures should be treated as indicative estimates from pilots and not guarantees for production.

**Adversarial robustness:** This study did not evaluate adversarial robustness, which remains an important concern. The hybrid architecture partially mitigates this through ML fallback; however, a dedicated adversarial analysis is required in future studies.

## 6 Conclusions

This study examined how a GenAI-based system fits into cybersecurity operations through a hybrid pipeline that combines traditional ML efficiency with the analytical and explanatory capabilities of the GenAI-based system.

**Key contributions:** The contributions are practical rather than theoretical: (1) systematic comparison showing the GenAI-based system has lower classification performance than ML but complementary capabilities; (2) empirical validation of confidence-based routing with threshold analysis justifying the selection of τ = 0.8; (3) proof-of-concept evidence for the GenAI-based system's explanation value, with human expert evaluation (*n* = 5, α = 0.78) indicating improvements over traditional methods, though the small sample limits generalizability; and (4) practical insights from pilot implementations.

**Practical implications:** Organizations considering GenAI integration should start with investigation support and explanations rather than autonomous decisions. Critical point: We found a 3% significant hallucination rate. This means that humans are required in the loop. All GenAI outputs should be validated by analysts before any action is taken. GenAI should help analysts and not make decisions independently. Our evidence suggests that GenAI adds value for complex, novel, or high-stakes events where ML confidence is low, but it should not replace ML for routine tasks or run without human oversight.

**Limitations:** Generalizability is constrained by several factors. The evaluation is based on two datasets, and neither fully captures the conditions of operational environments. GenAI performance on UNSW-NB15 (40% accuracy) also points to a basic limitation when language models are applied to largely numerical patterns. The case studies are controlled pilots rather than full production deployments. Cost remains a barrier as well, given the large computational gap between ML and GenAI. The expert panel is small (*n* = 5), so the results should be read as indicative and larger studies are needed. The evaluation also lacks two useful comparison points: a human-only baseline and stronger XAI baselines such as counterfactual explanations or concept bottleneck models, both of which would make the claims easier to support. One more limitation sits in the input representation. The feature-to-text step inevitably bakes in expert assumptions, meaning GenAI is reasoning over a narrated version of the features rather than the raw data itself. This limits what can be said about the model's intrinsic capabilities.

**Future research directions:** Several directions follow naturally from these limitations. One is the development of evaluation frameworks tailored to hybrid AI systems. Another is targeted fine-tuning to improve GenAI performance on numerical feature sets. Longitudinal studies in real deployments remain necessary to understand sustained utility and operational impact. Adversarial robustness of GenAI in security settings is also an open area that requires focused investigation.

**Concluding assessment:** The evidence points to a clear separation: traditional ML for automated, real-time detection; the GenAI-based system for assisted investigation with human oversight. This study advocates for carefully scoped human-in-the-loop augmentation, not autonomous GenAI detection. The practical value of GenAI-based systems in cybersecurity is not about outperforming established detectors; it is about improving how humans handle uncertain, novel, and high-stakes security decisions in the future.

## Data Availability

Publicly available datasets were analyzed in this study. This data can be found here: https://whyisyoung.github.io/BODMAS/.
