# Supplementary material for: Beyond performance metrics: evaluating the unique value of generative AI in hybrid cybersecurity threat detection
Source: Front Big Data. 2026 Apr 24;9:1768366. doi: 10.3389/fdata.2026.1768366 (PMC13157654; doi:10.3389/fdata.2026.1768366)
Supplement: Supplementary file 1 [file Supplementary_file_1.pdf]

Supplementary Material

1 SUPPLEMENTARY DATA

This Supplementary Material provides extended case study documentation, scalability analyses, and implementation details that support the main manuscript findings. Due to space constraints in the main text, detailed operational examples and architectural considerations are presented here.

2 S1: PROMPT TEMPLATES, FEATURE-TO-TEXT NARRATION RULES, AND PREPROCESSING DETAILS

2.1 Prompt Templates

The following prompt template was used for threat classification:

```
[SYSTEM] You are a senior cybersecurity analyst with expertise
in network intrusion detection. Analyze the following network
traffic sample and classify it as MALICIOUS or BENIGN. Provide:
1. Classification (MALICIOUS/BENIGN)
2. Confidence level (1-10)
3. Brief explanation of your reasoning
4. Key indicators that influenced your decision

[SAMPLE]
Connection: {source_ip}:{src_port} -> {dest_ip}:{dst_port}
Protocol: {protocol_name}
Duration: {duration}ms ({duration_context})
Bytes transferred: {bytes} ({volume_context})
Packet count: {packets} ({packet_context})
[Additional features as applicable]

[ANALYSIS]
```

2.2 Feature-to-Text Narration Rules

Each feature category was converted to natural language using the following templates:

- **Network flow metrics:** “Connection duration: {value} ms ({context: short-lived/long-lived/typical} connection)”
- **Protocol information:** “Protocol: {name} (commonly associated with {service description})”
- **Volume indicators:** “Bytes transferred: {value} ({context: low/moderate/high} relative to typical {protocol} sessions)”
- **Behavioral indicators:** “{feature name}: {value} ({deviation from baseline: normal/elevated/anomalous})”

Context annotations were derived from published network traffic baselines and standard service port mappings (IANA registry).

2.3 Data Preprocessing Details

The UNSW-NB15 preprocessing pipeline applied the following steps to reduce the original 2,540,043 samples to 2,300:

1. **Duplicate removal (67%):** Near-identical records were identified using feature vector similarity (cosine similarity > 0.99)
2. **Missing feature exclusion (28%):** Samples lacking features required for GenAI contextual analysis were removed
3. **Stratified sampling:** Class-balanced sampling ensured representation of all nine attack categories

Class Distribution Changes: The original UNSW-NB15 class distribution (87.4% Normal, 12.6% Attack) was modified to approximately 55% Normal, 45% Attack in our subset, intentionally over-representing attack classes to enable meaningful GenAI evaluation given prompt token constraints.

3 S2: ZERO-SHOT EVALUATION SCENARIO DESCRIPTIONS

The zero-shot evaluation (Section 4.2.1 of the main manuscript) used three synthetic scenarios representing emerging attack vectors. Each scenario was constructed from publicly documented attack techniques and presented to the LLM-based system without any prior training examples.

3.1 Scenario 1: AI-Generated Phishing Campaigns

This scenario simulated network traffic patterns associated with AI-generated spear-phishing campaigns. The traffic features were constructed to reflect: (1) automated email generation with dynamically varying sender profiles, (2) credential harvesting through cloned authentication pages with realistic SSL certificates, and (3) command-and-control callbacks using legitimate cloud service endpoints to evade domain-based filtering. The scenario was grounded in documented techniques from MITRE ATT&CK (T1566.001, T1598) and recent threat intelligence reports on LLM-assisted social engineering.

3.2 Scenario 2: IoT Botnet Command-and-Control Patterns

This scenario modeled communication patterns characteristic of IoT botnet infrastructure. Features included: (1) periodic beaconing with jittered intervals from multiple source IPs on non-standard ports, (2) DNS tunneling indicators with high entropy subdomain queries, and (3) lateral movement patterns across network segments with heterogeneous device profiles. The scenario drew on documented IoT botnet behaviors (Mirai variants, BotenaGo) and NIST SP 800-183 IoT threat models.

3.3 Scenario 3: Cryptographic Evasion Techniques

This scenario presented traffic patterns designed to evade traditional signature-based detection through advanced cryptographic techniques. Features included: (1) encrypted command channels using non-standard TLS configurations, (2) steganographic data exfiltration embedded in legitimate HTTPS sessions, and (3) protocol manipulation exploiting ambiguities in TLS 1.3 handshake extensions. The scenario was informed by academic literature on encrypted traffic analysis challenges and documented APT techniques.

Evaluation note: These scenarios were evaluated qualitatively by the expert panel (n=5). Assessments reflect expert plausibility judgments, not validated ground-truth performance measurements.

4 S3: HUMAN EVALUATION RUBRIC AND ANCHOR DEFINITIONS

The following standardized rubric was used by all five evaluators. Each GenAI explanation was rated independently on three dimensions using the 10-point scale defined below.

4.1 Clarity (1–10)

- 1–2: Incomprehensible or incoherent explanation
- 3–4: Major gaps; difficult to follow without significant effort
- 5–6: Adequate; conveys the main point but lacks detail or structure
- 7–8: Clear and well-structured; minor improvements possible
- 9–10: Exceptionally clear; immediately understandable by any SOC analyst

4.2 Actionability (1–10)

- 1–2: No actionable guidance; analyst cannot proceed based on the explanation
- 3–4: Vague guidance; analyst must conduct significant additional research
- 5–6: Some guidance; analyst can identify a general direction for investigation
- 7–8: Good guidance; analyst can begin specific response actions
- 9–10: Comprehensive actionable recommendations; analyst can proceed immediately

4.3 Usefulness (1–10)

- 1–2: Not useful in any realistic incident response workflow
- 3–4: Marginally useful; provides minimal added value over raw alert data
- 5–6: Moderately useful; saves some analyst time or effort
- 7–8: Useful; meaningfully improves the analyst's understanding of the incident
- 9–10: Highly valuable; substantially accelerates triage or escalation decisions

4.4 Evaluation Protocol

Each evaluator received 50 randomly selected GenAI explanations alongside: (1) the original network traffic features, (2) the ML classifier's prediction and confidence score, and (3) the ground truth label. Evaluators were blinded to each other's ratings. Inter-rater reliability was assessed using Krippendorff's alpha ($\alpha = 0.78$, ordinal variant). For explanations with high variance (SD > 1.5), a consensus discussion was conducted. Final scores for high-variance items reflect post-discussion consensus.
